# “Leopards do not change their spots:” tick borne disease symptomology case report

**DOI:** 10.1186/s12879-022-07683-x

**Published:** 2022-08-19

**Authors:** Haley Abernathy, Aidin Alejo, Victor Arahirwa, Odai Mansour, Amanda Brown-Marusiak, Dana Giandomenico, Ross M. Boyce

**Affiliations:** 1 Institute for Global Health and Infectious Diseases, University of North Carolina at Chapel Hill, 130 Mason Farm Road, MBRB 2336, CB 7030, Chapel Hill, NC 27599 USA; 2 School of Medicine, University of North Carolina at Chapel Hill, Chapel Hill, NC 27599 USA; 3 Department of Epidemiology, Gillings School of Global Public Health, University of North Carolina at Chapel Hill, Chapel Hill, NC 27599 USA

**Keywords:** Ehrlichiosis, Rocky Mountain Spotted Fever, Tick-borne disease, Case Report

## Abstract

**Background:**

Human Monocytic Ehrlichiosis is caused by infection with the bacteria *Ehrlichia chaffeensis* through the bite of an infected lone star tick (*Amblyomma americanum*). Patients infected with Human Monocytic Ehrlichiosis often present with symptoms including fever, headache, myalgia, and occasionally a macular rash. The presence of other endemic tick-borne diseases with similar symptoms, such as Rocky Mountain Spotted Fever, complicate the diagnosis of Human Monocytic Ehrlichiosis.

**Case presentation:**

A patient developed a fever, diffuse myalgia, headache, and a non-productive cough 5 days after a fishing trip in late May in central North Carolina. Over the course of the illness the patient’s symptoms worsened, with arthralgia, bilateral lower extremity erythema and edema, and a developing bilateral rash on the palms. With testing that revealed elevated liver enzymes, a potential for recent tick exposure (e.g., fishing trip), presentation during tick season, and the development of a rash, Rocky Mountain Spotted Fever and Human Monocytic Ehrlichiosis were considered. The patient was prescribed a seven-day course of oral doxycycline and cefalexin, which would provide coverage from *Rickettsia*, *Ehrlichia* and gram-positive bacteria typically responsible for cellulitis. Many of the patient’s symptoms resolved or improved, although the right shoulder remained painful to active movement. The patient was prescribed another seven-day course of doxycycline due to his perceived incomplete response to the first course. Approximately 5 weeks after symptom onset (D0 + 36), the patient followed up with a provider for convalescent testing and counseling. Convalescent *Ehrlichia* and *Rickettsia* serological tests were ordered. The acute *Ehrlichia* serology and acute *Rickettsia* serology were originally non-reactive with both titers measured at < 1:64. Convalescent serology, ordered 28 days after the acute sample collection, showed a greater than four-fold increase in the *Ehrlichia* IgG titer (1:256), satisfying clinical and laboratory case definitions for ehrlichiosis. In follow-up, 3 weeks later (D0 + 57), the patient reported that most of his pain had subsided, though he still occasionally got shooting nerve pain when exercising.

**Conclusion:**

This case of Human Monocytic Ehrlichiosis in North Carolina exemplifies the need for a knowledge of spatial epidemiological patterns and clinical manifestations in the diagnosis of tick-borne diseases.

## Case report

### Background

North Carolina experiences some of the highest rates of Rocky Mountain Spotted Fever (RMSF) and Human Monocytic Ehrlichiosis (HME) in the United States, often accounting for over 10% and 5% of national totals reported to the CDC, respectively [1]. Patients with HME frequently present with flu-like symptoms including fever, headache, and myalgia during the prodrome period. Up to 40% develop a macular rash, although this is more common in children [2]. In North Carolina, the presence of RMSF complicates the diagnosis and epidemiology of HME because the symptoms are difficult to distinguish, resulting in frequent under-recognition of HME despite evidence of similar infection rates [3]. The following report of a case of HME in North Carolina aims to highlight the importance of utilizing epidemiological patterns, laboratory data, and clinical presentations to accurately diagnose tick-borne diseases.

## Case presentation

**Fig. 1:**
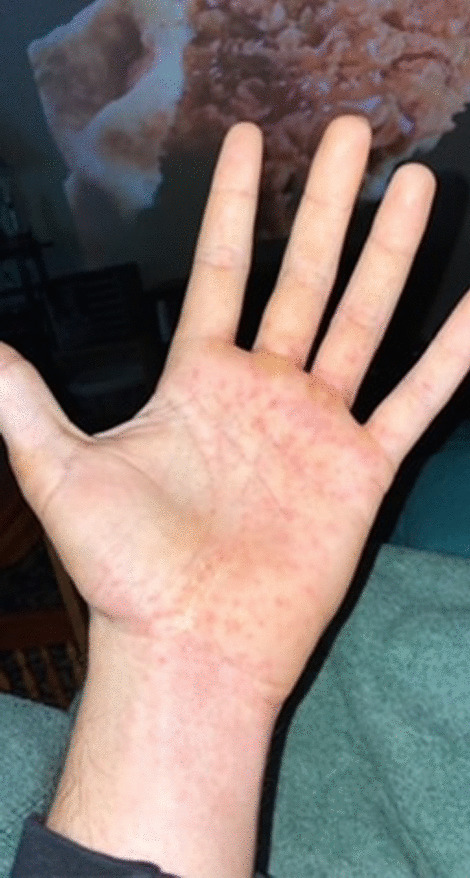
Nonpalpable bilateral rash on palms

**Fig. 2:**
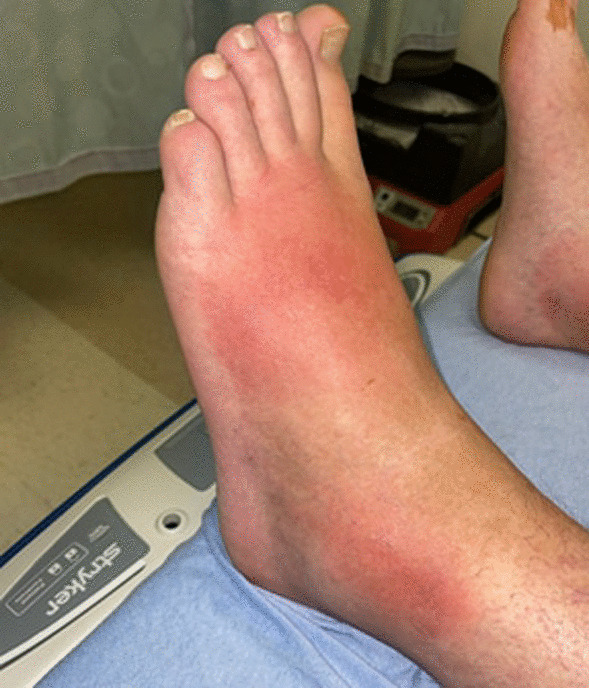
Patient presented with redness and pitting edema beginning 5 days after symptom onset (D0 + 5)

A 31-year-old man with a medical history notable for a traumatic spinal cord injury causing paraplegia developed a fever of 103° Fahrenheit (F), diffuse myalgia, headache, and a non-productive cough five days after (D0) a fishing trip in late May at a man-made reservoir in central North Carolina. Three days after symptom onset (D0 + 3), the patient sought care at a local urgent care where he tested negative for SARS-CoV-2 and influenza and received a presumptive diagnosis of an unspecified “viral illness.” He was advised to take over-the-counter medications for symptom management, stay hydrated, and follow up with his primary care physician (PCP). His symptoms progressively worsened, and a flat erythematous rash developed on the palms of both hands (Fig. 1). He returned to the urgent care five days after the initial visit (D0 + 8) with new bilateral lower extremity erythema and edema (Fig. 2). The provider was concerned for deep vein thrombosis (DVT) and advised the patient to go to a local emergency department (ED). In the ED, the patient’s vital signs included a blood pressure of 111/53 mm Hg, pulse of 86 beats per minute, temperature of 98.5° F, respiratory rate of 19 breaths per minute, and an oxygen saturation of 96%.

Results of complete blood count (CBC) were notable for a white blood cell count at the upper limit of normal (D0 + 5) with lymphopenia and a normocytic anemia (Table 1). A comprehensive metabolic panel (CMP) showed a slight elevation in his aspartate aminotransferase (AST), alanine transaminase (ALT), and alkaline phosphatase (ALP) levels. Erythrocyte sedimentation rate (ESR) and c-reactive protein (CRP) were also elevated at 79 mm/h and 342 mg/L, respectively. A urinalysis revealed moderately elevated leukocytes and trace ketones. Multiplex polymerase chain reaction (PCR) testing for influenza, respiratory syncytial virus, and SARS-CoV-2 was negative.

**Table 1:** Labs taken throughout patient’s illness

Labs	D0 + 5	D0 + 8	D0 + 9	D0 + 10	D0 + 11	D0 + 23	D0 + 36	Reference range
WBC	10.9	11.3	8.9	9.5				4.5–11 × 10^9/L
RBC	4.24	4.21	3.65	3.50				4.5–5.9 × 10^12/L
HGB	13.0	12.6	11.1	10.5				13.5–17.5 g/dL
HCT	39.4	39.0	34.0	32.4				41–53%
Platelet	295	352	391	367				150–440 × 10^9/L
Absolute neutrophils	9.7	10	7.2	7.8				2.0–7.5 × 10^9/L
Absolute lymphocytes	0.5	0.5	1.0	0.9				1.5–5.0 × 10^9/L
Hypochromasia	Slight (A)	Slight (A)	Slight (A)	Slight (A)				Not Present
Sodium	142	141	140	139	141	138	139	135–145 mmol/L
CO2	31	30	34	31	31	30.6	32.3	20–31 mmol/L
Creatinine	.81	.65	.69	.61	.69	.59	.69	.60–1.10 mg/dL
EGFR CKD-EPI	> 90	> 90	> 90	> 90	> 90	> 90	> 90	mL/min/1.73 m^2
Calcium	8.7	8.8	8.2	8.1	8.3	9.4	10.1	8.7–10.4 mg/dL
Albumin	3.5	3.2	2.5	2.5	2.7	2.7	4.0	3.4–5.0 g/dL
Total protein	6.4	6.0	5.1	5.1	5.4	6.8	7.9	5.7–8.2 g/dL
Globulin	2.9	2.8	2.6	2.6	2.7	4.1	3.9	2.0–3.5 g/dL
AST	37	137	164	124	156	62	68	< 34 U/L
ALT	58	134	169	161	180	131	144	10–49 U/L
Alkaline phosphatase	246	415	451	471	545	820	583	46–116 U/L

Abnormal values are red. CO2: Carbon Dioxide; BUN: Blood urea nitrogen; EFGR CKD-EPI: Estimated Glomerular Filtration Rate Chronic Kidney Disease Epidemiology Collaboration; AST: Aspartate aminotransferase; ALT: Alanine transaminases; WBC: White blood cell; RBC: Red blood cell; HGB: Hemoglobin; HCT: Hematocrit; MCV: Mean corpuscular volume; MCH: Mean corpuscular hemoglobin, MCHC: Mean corpuscular hemoglobin concentration; RDW: Red blood cell distribution width; MPV: Mean platelet volume

Values for absolute monocytes, absolute eosinophils, absolute basophils, large unstained cells, potassium, chloride, anion gap, BUN, BUN/creatinine ration, glucose, total bilirubin, MCV, MCH, MCHC, RDW, MPV, were within normal limits

The differential diagnosis for acute febrile illness is broad but is more limited when observed in conjunction with cutaneous skin manifestations and abnormal liver function tests. The normal work of breathing, stable oxygenation, unremarkable plain film of the chest, and negative testing for SARS-CoV-2 and influenza A/B reduced the likelihood of a pulmonary infection being the primary etiology. Human immunodeficiency virus (HIV) and syphilis were initially considered but ultimately did not align with patient symptoms. The presence of erythema and edema raised suspicion for DVT and cellulitis. The bilateral nature of his symptoms would be uncommon for these conditions and a negative venous duplex exam reduced the post-test probability of DVT.

The patient’s worsening fever and headache raised suspicion for infection, meningitis, and sepsis. Together with elevated liver enzymes, rash, a possible tick exposure (e.g., fishing trip), and the presentation during peak tick season [4], RMSF and HME were considered.

In the patient’s initial visit to the ED (D0 + 8), the resident physician recommended admission for further evaluation, but the patient preferred discharge with strict return precautions. He was prescribed a seven-day course of oral doxycycline and cefalexin, which would provide coverage from *Rickettsia*, *Ehrlichia* and gram-positive bacteria typically responsible for cellulitis.

Three days after discharge from the ED (D0 + 11), the rash on the patient’s palms and headache began improving, but his overall clinical condition worsened. He reported persistent fevers and worsening bilateral pain in his shoulders, wrists, and right middle finger. He returned to the ED where repeat laboratory testing was notable for a new leukocytosis, markedly elevated CRP and ESR as well as more pronounced elevations in his AST, ALT, and ALP (Table 1). The patient was continued on doxycycline, but also received fluid resuscitation with intravenous saline and broad-spectrum parenteral antibiotics (e.g., cefepime, vancomycin, and metronidazole). The attending physician recommended the patient be admitted to the hospital for sepsis.

During the admission several additional lab tests were performed for HIV, syphilis, gonorrhea, chlamydia, *Rickettsia rickettsii*, *Ehrlichia chaffeensis,* Lyme disease, parvovirus, toxoplasmosis, and Epstein-Barr virus (EBV). All were negative, except for EBV Viral Capsid Antigen IgG and EBV Nuclear Antigen IgG antibody, likely reflective of past infection [5]. An electrocardiogram and a chest X-ray were again unremarkable. The patient remained hospitalized for three days and doxycycline was continued after discharge for a total duration of seven days. At discharge (D0 + 14), the rash and fever had resolved, and the arthralgia was improving, although the right shoulder remained painful to active movement. The patient’s liver enzymes were still elevated and a follow up metabolic panel five days later showed a decrease in liver transaminase levels but an increase in ALP.

Six days after discharge (D0 + 20), the patient visited his PCP due to persistent joint pain and weakness in his right arm. Although not supported by current guidelines, the patient was prescribed another seven-day course of doxycycline due to his perceived incomplete response to the first course [4]. Hydrocodone was also prescribed for the pain. Six days later, the patient followed up with his PCP, reporting that his joint pain had significantly improved, and the fever had not returned.

Approximately 5 weeks after symptom onset (D0 + 36), the patient followed up with an infectious disease provider for convalescent testing and counseling. At that time, he endorsed feeling better and mentioned his energy was returning to baseline levels. The patient still had pain in his right shoulder, elbow, and wrist, suggestive of inflammation in the axillary and radial nerve distribution. Additionally, he commented that the skin on his palms had desquamated bilaterally.

Convalescent *Ehrlichia* and *Rickettsia* serological tests were ordered along with galactose-α-1,3-galactose (alpha-gal) IgE serology. The acute *Ehrlichia* serology and acute *Rickettsia* serology were originally non-reactive with both titers measured at < 1:64 during the hospital stay. Convalescent serology, ordered 28 days after the acute sample collection, showed a greater than four-fold increase in the *Ehrlichia* IgG titer (1:256), satisfying clinical and laboratory case definitions for ehrlichiosis. RMSF convalescent serology and alpha-gal IgE were both non-reactive. No further work-up was needed at the time. In follow-up 3 weeks later (D0 + 57), the patient reported that most of his pain had subsided, though he still occasionally got shooting nerve pain when exercising.

## Discussion and conclusion

The presence of a petechial rash on the distal extremities, including the palms and soles, is often considered pathognomonic for RMSF*.* In this case, however, while the rash was present on the patient’s palms, further characterization of the rash (e.g., blanching vs petechial) may have provided important clues about the diagnosis. Additional clues include the normal sodium concentration, normal platelet counts, and presence of a mild normocytic anemia, all of which are more characteristic of HME than RMSF [4]. Fortunately, doxycycline is first-line treatment for both diseases. This case highlights the importance of including ehrlichiosis in the differential of tick-borne infections, particularly in places where lone star ticks are the predominant vector. It also underscores the use of subtle differences in clinical and laboratory findings to guide diagnosis, especially when PCR testing and “gold standard” diagnostics require paired acute and convalescent samples that are not readily available as tick-borne diseases continue to spread into overlapping regions.

## Data Availability

Not applicable.

## Abbreviations

ALP: Alkaline phosphatase
ALT: Alanine transaminase
AST: Aspartate aminotransferase
BUN: Blood urea nitrogen
CBC: Complete blood count
CMP: Comprehensive metabolic panel
CO2: Carbon dioxide
CRP: C-reactive protein
DVT: Deep vein thrombosis
EBV: Epstein-Barr virus
ED: Emergency Department
EGFR CKD-EPI: Estimated Glomerular Filtration Rate Chronic Kidney Disease Epidemiology collaboration
ESR: Erythrocyte sedimentation rate
HCT: Hematocrit
HGB: Hemoglobin
HIV: Human immunodeficiency virus
HME: Human Monocytic Ehrlichiosis
MCH: Mean corpuscular hemoglobin
MCHC: Mean corpuscular hemoglobin concentration
MCV: Mean corpuscular volume
MPV: Mean platelet volume
PCP: Primary care physician
PCR: Polymerase chain reaction
RBC: Red blood cell
RDW: Red blood cell distribution width
RMSF: Rocky Mountain Spotted Fever
WBC: White blood cell

## References

[1] Centers for Disease Control and Prevention. National Notifiable Diseases Surveillance System, 2019 Annual Tables of Infectious Disease Data. CDC Division of Health Informatics and Surveillance. 2021.

[2] Paddock CD, Childs JE (2003). *Ehrlichia chaffeensis*: a prototypical emerging pathogen. Clin Microbiol Rev.

[3] Boyce RM (2018). Ehrlichia infections, North Carolina, USA, 2016. Emerg Infect Dis.

[4] Biggs HM, Behravesh CB, Bradley KK (2016). Diagnosis and management of tickborne rickettsial diseases: rocky mountain spotted fever and other spotted fever group rickettsioses, Ehrlichioses, and Anaplasmosis—United States. MMWR Recomm Rep.

[5] Klutts JS, Ford BA, Perez NR, Gronowski AM (2020). Evidence-based approach for interpretation of Epstein-Barr virus serological patterns. J Clin Microbiol.

